# Transgenic Hybrid Poplar for Sustainable and Scalable Production of the Commodity/Specialty Chemical, 2-Phenylethanol

**DOI:** 10.1371/journal.pone.0083169

**Published:** 2013-12-26

**Authors:** Michael A. Costa, Joaquim V. Marques, Doralyn S. Dalisay, Barrington Herman, Diana L. Bedgar, Laurence B. Davin, Norman G. Lewis

**Affiliations:** 1 Institute of Biological Chemistry, Washington State University, Pullman, Washington, United States of America; 2 Ealasid, Inc., Pullman, Washington, United States of America; Lawrence Berkeley National Laboratory, United States of America

## Abstract

Fast growing hybrid poplar offers the means for sustainable production of specialty and commodity chemicals, in addition to rapid biomass production for lignocellulosic deconstruction. Herein we describe transformation of fast-growing transgenic hybrid poplar lines to produce 2-phenylethanol, this being an important fragrance, flavor, aroma, and commodity chemical. It is also readily converted into styrene or ethyl benzene, the latter being an important commodity aviation fuel component. Introducing this biochemical pathway into hybrid poplars marks the beginnings of developing a platform for a sustainable chemical delivery system to afford this and other valuable specialty/commodity chemicals at the scale and cost needed. These modified plant lines mainly sequester 2-phenylethanol via carbohydrate and other covalently linked derivatives, thereby providing an additional advantage of effective storage until needed. The future potential of this technology is discussed. MALDI metabolite tissue imaging also established localization of these metabolites in the leaf vasculature.

## Introduction

There is an urgent need for sustainable, renewable, domestic sources of commodity/specialty chemicals, biofuels, and materials currently obtained from eventually dwindling petroleum and coal based resources at scale and cost both now and for our future generations. For example, several aromatics [e.g., styrenes, ethyl benzene, 2-phenylethanol (PEA)] are currently produced *synthetically* as commodity chemicals annually from our non-renewable and finite petrochemical resources [1]. Frequently, such processes have considerable drawbacks that include use of harsh synthetic conditions and production of undesirable side-products.

Naturally occurring PEA is a colorless, fragrant, liquid found in essential oils of several plant species. It has a pleasant “rose-like” aroma and is widely used as a fragrance and flavoring agent, as an antibiotic, and also for production of the flavoring agent phenylethyl-acetate [2]. Interestingly, synthetic and natural PEA substantially differ in value, with the synthetic product priced around US $3.5/ kg in the world market (∼7000 ton per year in 2007), while the natural biologically produced product, preferred for human consumption, can command prices up to US $1000/ kg, with a global estimated market in 2007 of 0.5–1 ton per year [3]. PEA is also readily converted into styrene [4] and ethyl benzene [5]. Currently, both styrene and ethyl benzene produced annually from petrochemical sources are in amounts of ∼4×10^6^ metric tons each in the US alone [1]. Ethyl benzene is a major aromatic component of aviation fuel.

Attempts to biotechnologically produce PEA in microorganisms have been made, but with no technical commercial success thus far. This is presumably because utilizing mainly the Ehrlich pathway of amino acid catabolism [3], [6], maximal productivity levels obtained in *S. cerevisiae* only reached ∼5 g/l and up to 25 g/l with *Kluyveromyces marxianus* [7]. However, this requires somewhat complex culture techniques that increase costs which make it economically unfavorable. Another limiting factor in microbial biotechnological PEA production is related to its inherent antimicrobial activity [2], [7], as it cannot be sequestered in stable (non-toxic) form or stored in a protective compartment (e.g., cell wall, vacuole, etc.) as frequently occurs for various natural products in vascular plants.

**Figure 1:**
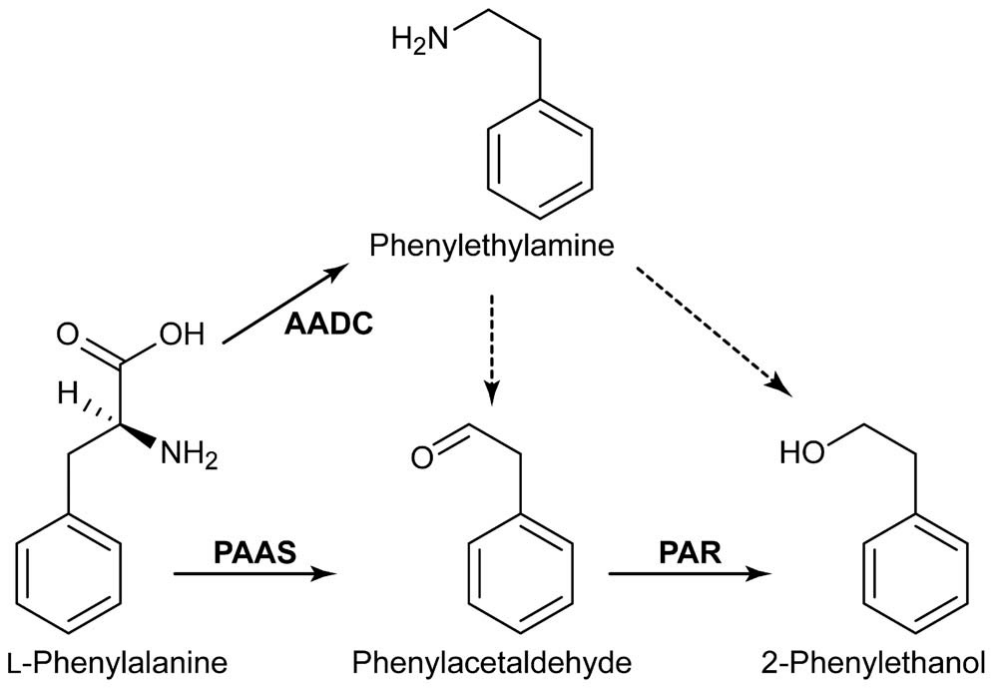
Possible biosynthetic pathways leading to 2-phenylethanol (PEA) production *in planta*. AADC aromatic amino acid decarboxylase; PAAS, phenylacetaldehyde synthase and PAR, phenylacetaldehyde reductase.

In plants, by contrast, PEA is apparently produced via two complementary biosynthetic pathways. The first consists of an initial decarboxylation, catalyzed by aromatic amino acid decarboxylase (AADC, Figure 1) [8], followed by an oxidative deamination (for which the corresponding enzyme/gene remain unknown). A second pathway consists of an initial step catalyzed by the bifunctional phenylacetaldehyde synthase (PAAS) that engenders sequential decarboxylation and oxidation of phenylalanine forming phenylacetaldehyde [9], with the latter reduced by phenylacetaldehyde reductase (PAR ) [10] to afford PEA (Figure 1). The putative function of PAAS was deduced from searching expressed sequence tag databases from both *Petunia hybrida* and *Rosa hybrida* for sequences similar to aminotransferases, decarboxylases and amine/monoamine oxidases [9]. As a result, two homologs to L-tyrosine/3,4-dihydroxy-L-phenylalanine (L-Dopa) decarboxylase were found, one from petunia and one from rose, having expression patterns correlated temporally and spatially with PEA production. Both were biochemically and functionally characterized *in vitro* as PAAS, bifunctional enzymes generating phenylacetaldehyde, CO_2_, ammonia and hydrogen peroxide in stoichiometric amounts. The gene encoding phenylacetaldehyde reductase (*PAR1*), a member of the large and diverse short-chain dehydrogenase/reductase family, was isolated/characterized from tomato (*Solanum lycopersicum*) [10].

We thus hypothesized that a rapidly growing woody biomass species might be modified biotechnologically for sustainably producing this highly valued chemical, and to begin to establish the potential of poplar as a biotechnological platform for production of specialty and commodity chemicals. In this context, hybrid poplar (*Populus tremula*×*Populus alba*) produces large amounts of biomass in a relatively short time period, and its fast growth, rapid propagation, and regenerative ability [11], [12] to form coppice shoots allow for multiple harvests per lifespan. Its transformation efficiency and regeneration also lend it suitable to expedient genetic engineering strategies [12].

Indeed, many woody plant species can accumulate various low molecular weight metabolites in their (heartwood) tissues, with aromatics in total accumulating to up to *circa* 60% [13]. This suggests that various biotechnological manipulations might offer a new strategy to obtaining desired specialty/commodity chemicals at scale and cost. The goal herein was therefore to initially demonstrate that the biochemical pathway to PEA could be introduced through heterologous expression strategies of both PAAS and PAR (Figure 1) as a proof-of-principle. We also envisaged the possibility of such metabolites being sequestered in non-volatile, non-toxic, forms *in planta* via derivatization (e.g., glycosylation, acylation, etc.), and thus enabling a means of continued production and storage until needed.

Initially, we carried out expression of PAAS and PAR in *Escherichia coli* to establish whether this coupled enzyme system would afford PEA, as there were no studies previously with both enzymes used in tandem. These same gene combinations were then over-expressed in hybrid poplar. Results from our expression studies are reported herein, as well as a discussion of the future potential of these technologies.

## Materials and Methods

### Plant Materials

Hybrid poplar *Populus tremula*×*P. alba* (INRA clone 717 1-B4, female) was obtained from Prof. Steven H. Strauss (Oregon State University) and was maintained in greenhouse conditions under 16 h day supplemental lighting with high pressure sodium lamps, at a temperature of 24°C day/21°C night and with 40–60% relative humidity. Sterile shoot cuttings from wild type plants were propagated and maintained in our tissue culture facility in sterile agar medium [14] with 16 h light and an ambient temperature of 22°C.

*Rosa hybrida* cv. Fragrant Cloud Hybrid Tea stock used for cDNA preparation was obtained from Jackson & Perkins (Hodges, SC 29653) as a bareroot plant and maintained in greenhouse conditions under natural lighting at a temperature of 24°C day/18°C night with 40–60% relative humidity.

*Petunia hybrida* cv. Mitchell plants used for cDNA preparation were produced from seed obtained from Michelle Jones (The Ohio State University, Horticulture and Crop Science, Ohio Agricultural Research and Development Center, Wooster, OH) and were maintained in a growth chamber under a 16 h day supplemental lighting at 200 µmol with fluorescent and incandescent lighting, at a temperature regime of 25°C day/18°C night and with 70–80% relative humidity.

### RNA Extraction and cDNA Preparation

Total RNA was individually isolated from 100 mg of flash-frozen petal limb tissue of a partially open stage 4 rose bud [15], early-stage flowering petunia corolla limbs, and tomato young leaf, respectively, using a SPECTRUM PLANT TOTAL RNA KIT (Sigma) according to the manufacturer’s instructions. After DNAse I treatment (Deoxyribonuclease I, Amplification Grade (Invitrogen)), first strand cDNA synthesis was individually performed for each plant tissue using an aliquot (1 µg) of the total RNA with a SuperScript III First-Strand Synthesis System for RT-PCR (Invitrogen) according to the manufacturer’s instructions.

### Gene Cloning

The *RhPAAS* and *PhPAAS* genes were amplified from rose petal and petunia corolla limb cDNA using the primers RhFCPAAS-F and RhFCPAAS-R, or PhMPAAS-F and PhMPAAS-R, respectively (Table S1), designed from the published cDNA sequences for *RhPAAS* (GenBank accession number DQ192639) and *PhPAAS* (GenBank accession number DQ243784) [9]. The *PAR1* gene was amplified using primers LePAR1-F and LePAR1-R (Table S1) designed based on the published cDNA sequence (GenBank accession number EF613490) [10]. Amplification was performed using *Pfu Turbo* DNA Polymerase (Stratagene) in a PCR mix containing 1 µl of the cDNA preparation. PCR amplification was accomplished using a Bio-Rad C1000 Thermal Cycler, with an initial denaturation step of 95°C for 2 min, followed by 35 cycles of 94°C denaturing for 30 s, 55°C annealing for 30 s, and 70°C extension for 3 min, with a final extension at 70°C for 10 min. PCR products were resolved using UltraPure low melting point agarose gel (Invitrogen), where a single band of approximately 1500 bp was obtained for each of the two *PAAS* genes and of 1000 bp for the *PAR1* gene. The *RhPAAS*, *PhPAAS*, and *PAR1* gene products purified from the PCR gel were cloned into either a pENTR/D-TOPO or pCR4–TOPO vector (Invitrogen) for sequence confirmation.

### Heterologous Expression in *E. coli*

*RhPAAS* and *PAR1* genes were cloned in tandem into a pETDuet-1 expression vector (Novagen) as follows. *PAR1* cDNA was first PCR amplified using primers PAR1-Nde I-DUET-FOR and PAR1-Xho I-DUET-REV (Table S1) to include *Nde* I and *Xho* I restriction enzyme sites at the 5′- and 3′-ends, respectively, then cloned into a pCR4-TOPO vector and excised and then cloned into the MCS2 region of the pETDuet-1 vector. The *RhPAAS* cDNA was PCR amplified with primers that included *BspH* I and *Not* I restriction enzyme sites (RhFCPAAS-BspH I-DUET-F and RhFCPAAS-Not I-DUET-R (Table S1)) and was cloned into a pENTR/D-TOPO vector. The adapted *RhPAAS* was then cut out from the pENTR/D-TOPO vector construct using the *BspH* I and *Not* I restriction enzyme sites and ligated into the compatible *Nco* I and *Not* I sites in the MCS1 region of the previously prepared *PAR1*/pETDuet-1 construct. A similar cloning scheme was used to incorporate the *PhPAAS* cDNA into the *PAR1*/pETDuet-1 construct, except that the PCR primer adapter restriction enzyme cloning sites to clone into the MCS1 were *Bsp*H I and *Sac* I (PhMPAAS-BspH I-DUET-F and PhMPAAS-Sac I-DUET-R (Table S1)), respectively. Competent BL21 (DE3) singles *E. coli* cells (Novagen) were individually transformed with 20 ng of the final plasmid preparations. With the empty pETDuet-1 vector used as a control, 50 ml LB/carbenicillin (100 µg/ml) cultures of each expression vector assembly were incubated at 37°C to an OD_600_ of 0.8. These were induced with isopropyl β-D-1-thiogalactopyranoside (IPTG) to a final concentration of 1 mM, in the presence of phenylalanine (5 mM), then sampled and pelleted over a 50 h time frame, with the supernatants individually extracted with hexane (1 ml) containing 0.5 mM benzyl-methyl-ether as internal standard and analyzed directly via GC-MS for presence of PEA (see Figure S1).

### Vector Construction for Over-expression in Hybrid Poplar

The 1.5 kb *RhPAAS* and *PhPAAS* cDNAs were next individually amplified with primers that included 5′-*Xho* I or 3′-*Bgl* II restriction enzyme sites for subsequent cloning into the pART7 vector [16] (Table S1, primers RhFCPAAS-XHOI-FOR and RhFCPAAS-BGLII-REV, or PhMPAAS-XHOI-FOR and PhMPAAS-BGLII-R, respectively). The PCR products from each were separated on and eluted from an UltraPure low melting point agarose gel and were again cloned into pENTR/D-TOPO vector. Following sequencing confirmation, the *RhPAAS* and *PhPAAS* cDNA were cut out using a double digest of *Xho* I and *Bgl* II restriction endonucleases and, after gel purification, ligated into the pART7 vector in the MCS region *Xho* I and *BamH* I sites.

A new set of primers was designed to amplify the fragment of the pART construct to include the CaMV 35S promoter plus the *RhPAAS* cDNA and the OCS terminator, with adapters on each end for use in a BP Clonase recombination with pDONR221 P1-P5r vector (Table S1, primers (PAAS)-attB1-p35S-F and (PAAS)-attB5r-tOCS-R). The BP Clonase reaction was carried out according to the Invitrogen protocol for a Gateway pDONR Recombination Reaction and included 3 µl attB PCR product, 1 µl pDONR221 plasmid, and 2 µl BP Clonase II enzyme mix. This procedure was also performed using the same two primers above to amplify the *PhPAAS* cDNA bordered by the 35S promoter and OCS terminator in the respective pART7 construct and the corresponding PCR product was then recombined into the pDONR221 P1-P5r vector.

The *PAR1*/pENTR/D-TOPO construct was incorporated into the Gateway binary vector pK2GW7 for overexpression using Gateway LR Clonase II (Invitrogen). Primers designed to amplify the p35S::*PAR1*::t35S fragment from the *PAR1*-pK2GW7 construct (Table S1, primers (LePAR1)-attB5-p35S-F and (LePAR1)-attB2-t35S-R) included adapter sequences for BP Clonase recombination into the pDONR221 P5-P2 vector (Invitrogen).

The *RhPAAS*-pDONR221 P1-P5r, or the *PhPAAS*-pDONR221 P1-P5r, and *PAR1*-pDONR221 P5-P2 constructs were subsequently cloned in a two-fragment MultiSite Gateway Pro Plus Kit (Invitrogen) recombination scheme [17] into the promoterless Gateway binary vector pKGW (http://gateway.psb.ugent.be) with a Gateway 2-Fragment recombination reaction using LR Clonase II Plus enzyme (Invitrogen) according to the manufacturer’s instructions. The final recombined construct was transformed into *E. coli* and plated onto LB/spectinomycin (50 µg/ml) medium.

All plasmids were prepared using the Wizard Plus SV Minipreps DNA Purification System (Promega), and these were propagated in One Shot Mach1-T1 Chemically Competent *E. coli* (Invitrogen). Primers used for PCR and for sequencing were ordered from Invitrogen. Samples for DNA sequencing were submitted to Eurofins MWG Operon SimpleSeq DNA sequencing service. The final Gateway constructs propagated in *E. coli* were subjected to DNA sequencing to confirm correct insertion of all gene fragments-of-interest in the T-DNA binary vector, with these then introduced into the *Agrobacterium tumefaciens* strain LBA4404 using a freeze-thaw method [18]. Transformed *A. tumefaciens* colonies were screened by PCR using appropriate transgene and vector specific primer combinations to confirm that the correct T-DNA vectors had been assimilated. Gene specific primers for Real-Time qPCR expression analysis were designed using the PRIMER PREMIER software program (Biosoft International, Palo Alto, CA, USA).

### Poplar Transformation

Hybrid poplar (*Populus tremula*×*P. alba*) stem and leaf pieces were transformed by co-cultivation with *A. tumefaciens* containing either the *RhPAAS/PAR1/*pKGW or the *PhPAAS/PAR1/*pKGW constructs, or an empty binary expression vector control, using methods similar to those of Ma *et al*. [14]. Shoots were generated and selected on solid agar medium (Agar-TC, Phytotechnologies, Inc.) containing kanamycin sulfate (GIBCO by Life technologies) at a final concentration of 100 µg/ml. Transformed shoots were identified initially by survival and growth on medium containing kanamycin as the selectable marker. Genomic DNA from regenerated plants was assayed directly by PCR screening using a REDExtract-N-Amp Plant PCR Kit (Sigma) to confirm the stable integration of the T-DNA containing the transgenes of interest and the neomycin phosphotransferase II gene present in all transformants, including those containing the empty vector T-DNA, into the poplar genome. After a three month selection process, plants that contained the introduced genes-of-interest were then transferred to the greenhouse.

### Real-time qPCR Analysis

Leaves (third from the apex) from 7 week old (after transfer to greenhouse) hybrid poplars were harvested, flash frozen and individually pulverized. Total RNA was prepared using the SPECTRUM PLANT TOTAL RNA KIT (Sigma). After DNAse I treatment (Deoxyribonuclease I, Amplification Grade (Invitrogen)), an aliquot (2 µg) of the total RNA for each specimen was subsequently used to prepare cDNA by performing RT-PCR using the SuperScript III First-Strand Synthesis System for RT-PCR (Invitrogen). Gene amplification was performed using QPCR Platinum SYBR Green qPCR SuperMix-UDG (Invitrogen) for the RT-qPCR reactions. The reaction mix included forward and reverse gene-specific primers, buffer, and ROX Reference Dye. A Stratagene Mx3005p QPCR System was used for real-time qPCR gene amplification. The internal reference gene control was an *actin* gene isolated from hybrid poplar. Primer sequences used for real-time qPCR are shown in Table S1 as RhFCPAAS-QPCR-F and RhFCPAAS-QPCR-R for the *RhPAAS*, PhMPAAS-QPCR-F and PhMPAAS-QPCR-R for *PhPAAS*, LePAR1-QPCR-F and LePAR1-QPCR-R for *PAR1*, and PtaACTIN-QPCR-F and PtaACTIN-QPCR-R for the reference *actin* gene. After an initial screening of transgenic plants compared to a wild type plant, a transgenic plant expressing the lowest levels of *RhPAAS*, or *PhPAAS*, and *PAR1* genes compared to the wild type plant was selected as the control calibrator in all future real-time qPCR comparisons of the respective transgenes. The real-time qPCR reactions were performed in triplicate using 2 µl of a 1∶5 dilution of the First-Strand cDNA preparation.

### Metabolite Extraction and Analysis

Leaves (from the third apical and basal internodes) and stems were collected from 7 weeks and 4 months old hybrid poplars. Plant materials in triplicate biological replicates were flash frozen individually in liquid nitrogen, ground using a 5-mm steel ball in a Tissue-lyzer (Qiagen) still frozen and kept at –80°C until extraction. For free PEA analyses, samples were extracted with methyl-*tert*-butyl ether containing 0.5 mM benzyl-methyl-ether as internal standard (2 µl/mg tissue) and extracts were individually analyzed using an HP 6890 Series GC System equipped with a RESTEK-5Sil-MS (30 m×250 mm×0.25 mm) column. The temperature program used was as follows: 40°C maintained for 2 min and raised from 40 to 150°C at 10°C/min, then from 150 to 250°C at 20°C/min with a final holding time of 2 min; total run time 22 min. Injector and detector temperatures were set at 250 and 230°C, respectively. PEA was quantified based on *m/z* 122 and 91 extracted ion traces and areas normalized to benzyl-methyl-ether peak area and quantified using external calibration with authentic PEA standard. Each chemical analysis data point is the average of three independent transgenic lines. The internal codes used were 1394 and 1395 for the empty vector lines; 1338, 1383 and 1384 for the *PhPAAS/PAR1* lines and 1336, 1337, and 1377 for the *RhPAAS/PAR1* lines. The real-time qPCR measurements and metabolite measurements were performed on samples from the same trees.

For 2-phenylethanol glucoside (PEA-Glc) determination, samples were extracted with methanol:water (7∶3, v/v) containing 0.5 mM naringenin as internal standard (10 µl/mg tissue) and analyzed using a Waters Acquity ultra performance liquid chromatography (UPLC) system equipped with a Waters BEH C18 column (1.7 µm particles, 2.1×100 mm) with a binary mobile phase of 0.1% formic acid in water (A) and 0.1% formic acid in acetonitrile (B), with detection via electrospray ionization mass spectrometry in the positive mode in a Waters Xevo G2 Q-TOF mass spectrometer using as a lock-mass standard leucine–enkephalin, capillary voltage of 3 kV, cone voltage of 38 V, desolvation gas temperature of 280°C and source temperature of 100°C. The gradient program was as follows: flow rate of 0.2 ml/min; linear gradient of A:B from 95∶5 to 65∶35 in 35.5 min, to 0∶100 in 1 min, followed by 2 min at 0∶100 and re-equilibration at initial conditions for 3 min. The column temperature was held at 25°C and sample injection volume was 1 µl.

Detection and quantification of PEA-Glc was performed, using external calibration with synthetic authentic PEA-Glc standard [19], by integrating the corresponding peak with retention time of 10.7 minutes in the extracted *m/z* 307.116 (corresponding to [M+Na]^+^ ) chromatogram. Peak areas were corrected to the peak area of the internal standard naringenin, from the extracted *m/z* 273.076 ([M+H]^+^).

The LCMS data generated was later analyzed using XCMS and CAMERA R packages to attempt to detect unforeseen effects of the transgenes in overall metabolism (i.e., downstream products from the introduced pathway and systemic changes in metabolite pools). Raw data was thus initially converted into NetCDF format using DataBridge software, for subsequent pairwise comparison between the high producing PEA/PEA-Glc transgenic poplar and those transformed with the empty vector in the XCMS R package (http://metlin.scripps.edu/download/), and then processed through the CAMERA package (http://bioconductor.org/packages/2.12/bioc/html/CAMERA.html) for peak groups assignment and mass spectra deconvolution for differentially accumulated metabolites. XCMS peak detection was performed using the Centwave method with the following parameters: prefilter = c(0,0), snthr = 3, ppm = 15, peakwidth = c(5,20), and nSlaves = 4. Retention time correction was achieved in two iterations using the obiwarp method and the following parameters: bw = 2, minfrac = 0.5, mzwid = 0.015 and plottype = c(‘deviation’). Peak group annotation using CAMERA was performed using the following parameters: perfwhm = 0.6 and groupCorr, findIsotopes and findAdducts with polarity = ’positive’. Data analysis thus generated was manually analysed and putative annotation performed using accurate mass, known fragmentation patterns and literature described mass spectra of known compounds, previously identified in poplar species.

### MALDI-TOF Imaging Mass Spectrometry (IMS) on Leaf Tissues of Transgenic Poplars

For MALDI-TOF IMS, fresh leaves of transgenic poplars were securely placed on a MALDI target plate using double-sided tape (3M). The fresh leaves were dried in a vacuum desiccator for 60 min prior to application with the matrix. Two MALDI matrices, α-cyano-4-hydroxycinnamic acid (30 mg/ml, Sigma) and 2,5-dihydroxybenzoic acid (DHB, 30 mg/ml, Sigma) in 50∶50 (v/v) methanol:water were tested to establish which was best for detection of synthetic PEA-Glc after uniform application on the MALDI target plate by an airbrush spraying device (Harder and Steenbeck Infinity Solo, Germany). The coated leaves were dried in a vacuum desiccator for 60 min before analysis. Optical images of the leaves were taken using a standard flatbed scanner (Epson Perfection V500 photo, Japan). MALDI IMS experiments were carried out using the Synapt G2 HDMS (MALDI Q-TOF MS, Waters Inc., UK). The instrument was calibrated in positive mode with red phosphorus (10 mg/ml in acetone). The data were acquired in positive mode using resolution mode in the range *m/z* 100 to 700 Da with spatial resolution of 50 µm at laser energy of 250 and firing rate of 1000 Hz. The area selected for imaging was defined using HDI imaging software (v1.1, Waters Inc.).

There were about 30,000 laser shots per leaf sample at a sampling rate of 0.5 sec per pixel. The raw data was then processed and ion maps were visualized in HDI™ imaging software. The one hundred most intense peaks were extracted using a mass filter of 0.02 Da, mass resolution of 10,000 and low energy intensity threshold of 10,000. The mass filter was kept constant on different samples and tissues. Collision-induced dissociation MS/MS of PEA-Glc (precursor ion, *m/z* 323.09 [M+K]^+^) and tremuloidin (precursor ion, *m/z* 429.09 [M+K]^+^) in leaf of trees transformed with *RhPAAS/PAR1* was performed at collision energy of 90 V (trap) and 45 V (transfer).

## Results

Both rose and petunia PAAS were used to transform hybrid poplar, in order to gauge and assess each of their relative efficacies for PEA production *in vivo.* Accordingly, the *PAAS* gene was cloned from both *R. hybrida* (cv. Fragrant Cloud) petals (*RhPAAS*) and *P. hybrida* (cv. Mitchell) corolla limbs *(PhPAAS*), whereas the *PAR1* was obtained from *S. lycopersicum* young leaf tissue (see Materials and Methods section).

### Phenylethanol Production in *E. Coli*

We initially expressed both *PAAS* genes simultaneously with the tomato *PAR1* in an *E. coli* system for proof-of-concept that their synchronized gene expression would result in PEA production. Each *PAAS* was thus individually cloned in tandem with the *PAR1* gene into a pETDuet vector that provided for synchronized expression of both *PAAS* and *PAR1* upon induction in an *E. coli* host. *E. coli* transformed with either *RhPAAS/PAR1* or *PhPAAS/PAR1* was thus grown in the presence of phenylalanine (5 mM). After induction by IPTG, successful PEA production was achieved in both lines. Culture medium concentration reached circa 5 and 8 µM for the petunia and rose *PAAS* genes, respectively, after 50 hour cultivation. Phenylacetaldehyde accumulation, on the other hand, reached respectively only 0.4 and 0.6 µM, (Figure S1). Nevertheless, with overall biochemical pathway effectiveness demonstrated, we next proceeded to poplar transfection.

### Phenylethanol Production in Hybrid Poplar

Leaf pieces from sterile 8-week-old hybrid poplar plants were co-cultivated for one hour with gentle shaking in a suspension of *Agrobacterium* strain LBA4404 containing either the *RhPAAS/PAR1* or the *PhPAAS/PAR1* gene combinations in pKGW binary expression vectors. Following a two-day incubation on solid callus-producing medium with no antibiotics, the leaf pieces were then transferred to the same medium but now containing the selectable marker antibiotic kanamycin, in addition to ticarcillin for elimination of the *Agrobacterium* following transfection.

**Figure 2:**
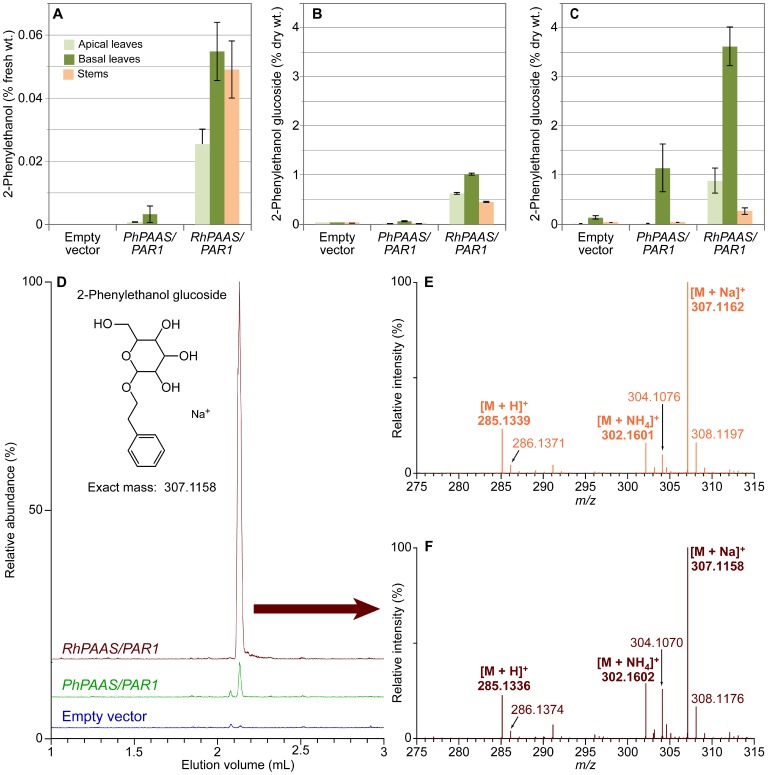
2-Phenylethanol (PEA) and its glucoside (PEA-Glc) accumulation in transgenic hybrid poplar. (A) 2-Phenylethanol and (B) 2-phenylethanol glucoside contents in leaves and stems from 7 week old hybrid poplar. (C) 2-Phenylethanol glucoside contents in leaves and stems from 4 month old hybrid poplar. (D) UPLC-MS relative abundances of PEA-Glc (*m/z* [M+Na]^+^) from 7 week old hybrid poplar transformed with empty vector, and those transformed with *PhPAAS/PAR1* and *RhPAAS/PAR1*, respectively. Mass spectra from authentic standard PEA-Glc (E) and from *RhPAAS/PAR1* leaf tissue (F). Error bars in figures A, B and C represent standard deviation, with n = 3.

Poplar co-cultivation with *Agrobacterium* containing the *RhPAAS/PAR1* or *PhPAAS/PAR1* constructs produced numerous shoots from callus tissue within 8 weeks. Genomic PCR screening then indicated that nearly all regenerated shoots selected on kanamycin contained the transgenes of interest. At this stage of growth/development, the transgenic poplar plants also appeared to grow normally with no obvious phenotypic alterations compared to wild type or control plants transformed with *Agrobacterium* containing the empty binary vector. Interestingly, from an early shoot development stage, while the shoots were still in agar medium in enclosed sterile containers, a distinct rose floral scent was noticeable emanating from the transgenic plants, suggesting PEA production. Seven week old trees (after rooted plantlets were transferred to soil) transformed with either the *RhPAAS*/*PAR1* or *PhPAAS*/*PAR1* constructs had distinct but somewhat opposite transcript levels of both *PAAS* and *PAR1* (Figure S2). To analyse these for PEA content, leaves (from the third apical and basal internodes) and stems were individually collected, with PEA levels determined as described in the Materials and Methods section. As demonstrated by GC-MS analyses, the transgenic hybrid poplar lines were able to produce and accumulate PEA in leaves and stems (Figure 2A). Amounts of PEA in *RhPAAS*/*PAR1* transformants were ∼0.05% fresh weight in old leaves/stems, and ∼17 fold lower in *PhPAAS*/*PAR1* transformants where levels reached only 0.003%.

Additional LC-ESI-MS metabolomic analyses established a much higher accumulation of the related 2-phenylethanol glucoside (PEA-Glc) in both of these tissues relative to PEA (Figure 2B). This was identified and quantified as having the same retention times (Figure 2D) and mass spectra (Figure 2F) by comparison with an authentic standard (Figure 2E). PEA-Glc from the *RhPAAS*/*PAR1* transformants had a protonated ion [M+H]^+^ of *m/z* 285.1336 (calc. 285.1338), ammonium adduct [M+NH_4_]^+^ of *m/z* 302.1602 (calc. 302.1604), and a sodiated ion [M+Na]^+^ of *m/z* 307.1158 (calc. 307.1158) (Figure 2F). As indicated above for PEA, the levels of PEA-Glc were also substantially lower (by ∼50, 20 and over 100 fold in younger leaves, older leaves and stems, respectively) in the *PhPAAS*/*PAR1* transformants at 7 weeks of growth and development (Figure 2B and D). An increased accumulation of PEA-Glc was observed, however, as plants matured, i.e., leaves from the basal node of 4-month-old trees had reached levels of 3–4% dry weight, while in stems, the levels had increased to *circa* 0.3% at this stage of growth and development (Figure 2C). The LCMS data obtained for leaves and stems was also subjected to an untargeted metabolite analysis using specialized software XCMS and CAMERA. The first software performs unsupervised retention time correction and peak integration in the three dimensional *m/z*/retention time/intensity space, followed by pairwise comparison between samples with appropriate statistical analysis. The R package groups together masses observed with the same chromatographic behavior and searches for known ions and fragmentations (e.g., protonated and sodiated ions, water and hexose losses). From this analysis, we were able to determine that, besides PEA-Glc being produced, other putative 2-phenylethanol derivatives (annotated based on accurate mass and fragmentation pattern) also accumulated. Interestingly, this was accompanied by a decrease in accumulation of other metabolites putatively annotated as phenylalanine-derived compounds known to be produced in *Populus* species [20] (i.e., glycosylated flavonoids and phenylpropanoids) relative to the empty vector line (Table S2).

### MALDI-TOF Imaging Mass Spectrometry

**Figure 3:**
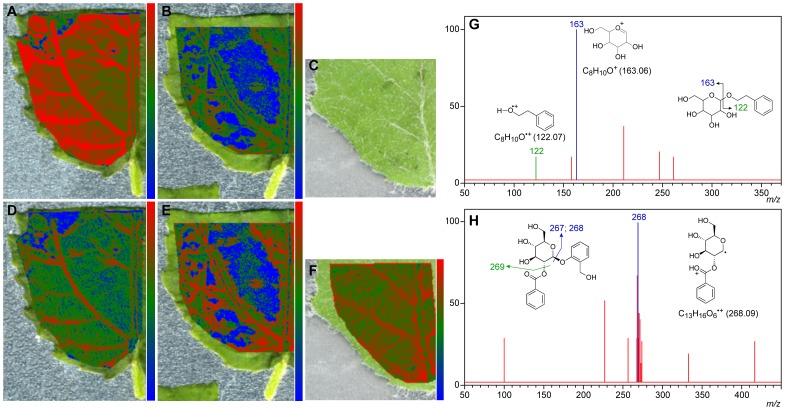
Spatial distribution of PEA-Glc and tremuloidin in leaf tissues of hybrid poplar as analyzed by MALDI imaging mass spectrometry. MALDI image and ion intensity map of PEA-Glc (*m/z* 323.093 [M+K]^+^) (A–C) and tremuloidin (*m/z* 429.096 [M+K]^+^) (D–F) in hybrid poplar transformed with: *RhPAAS*/*PAR1* (A and D), *PhPAAS*/*PAR1* (B and E), and an empty vector (C and F), respectively. Data was obtained with spatial resolution of 50 µm and using 2,5-dihydroxybenzoic acid as matrix. Note: The midrib was resistant to laser ablation and thus detection of metabolites was apparently not achieved fully in this region. Collision-induced dissociation MS/MS fragmentation of (G) PEA-Glc (*m/z* 323.093 [M+K]^+^) and (H) tremuloidin (*m/z* 429.096 [M+K]^+^). CID fragmentation was performed at a collision energy of 90 V (trap) and 45 V (transfer).

The spatial localization of PEA-Glc *in situ* in leaves of transgenic hybrid poplar lines was next mapped using matrix-assisted laser desorption/ionization-time of flight imaging mass spectrometry, MALDI-TOF IMS. With synthetic PEA-Glc as standard, ions *m/z* 307.114 and 323.093 corresponding to [M+Na]^+^ and [M+K]^+^, respectively, were readily detected and identified when using 2,5-dihydroxybenzoic acid (DHB) as matrix (data not shown). Next, *RhPAAS/PAR1* and *PhPAAS/PAR1* transformant poplar leaf sections were examined. This established the presence of hundreds of metabolites ranging from *m/z* 100 to 700 Da, with the most intense and abundant peak detected being the K^+^ adduct ion (*m/z* 323.093) of PEA-Glc (e.g., Figure S3A). This metabolite is abundantly distributed throughout the leaf, being concentrated mainly in the vasculature of *RhPAAS/PAR1* (Figure 3A), and to a lesser extent in the same regions in *PhPAAS/PAR1* (Figure 3B). By contrast, while the *PhPAAS/PAR1* transformant leaf also had hundreds of metabolites with mass ranges between *m/z* 100 to 700 Da (Figure S3B), PEA-Glc [M+K]^+^ was apparently less intense (*ca.* 9 fold decrease) relative to the *RhPAAS/PAR1* transformant leaf (Figure S3A). Additionally, the leaf from hybrid poplar transformed with an empty vector had hundreds of metabolites (Figure S3C), but none corresponded to PEA-Glc (Figure 3C). These results are thus in agreement with the data acquired from the UPLC-MS analysis of the crude leaf extracts (Figure 2B), with the latter analyses permitting comparisons of relative levels of PEA-Glc in the different lines. Collision-induced dissociation MS/MS analysis was also performed on the potassiated adduct ion [M+K]^+^ at *m/z* 323.093. Its spectrum had fragments at *m/z* 163 and 122 (Figure 3G), which represent the glucose carbocation and the radical cation aglycone, respectively, thus making an unequivocal identification of PEA-Glc in the *RhPAAS/PAR1* transgenic leaf. Interestingly, based on the experimental conditions employed/applied thus far, the midrib was resistant to analysis of these metabolites using laser ablation, even at higher laser energy (data not shown). However, we were able to establish they were present as well in the midrib by means of individual extraction of the midrib and lamina tissues separately (data not shown).

We were also able to detect and localize three known poplar phenolic glycosides (tremuloidin, salicin and salicortin) [21]–[23] (Figure S3, 3D–F, and S4). Similar to PEA-Glc, tremuloidin (*m/z* 429.096 [M+K]^+^) is more localized in the veins (Figure 3D and E) of the leaf tissue. Furthermore, it is apparently more abundant in the leaf vasculature of hybrid poplar transformed with an empty vector (Figure 3F), whereas it was seemingly less abundant in the same cell types of the *RhPAAS/PAR1* and *PhPAAS/PAR1* transgenic poplar leaves based on lower signal intensity counts, respectively (Figure 3D and E). This was confirmed by LCMS analysis which indicated an ∼1.3 fold reduction in its amount in the leaf tissue (see Table S2). Collision-induced dissociation MS/MS analysis was also performed on the potassiated adduct ion at *m/z* 429.096, [M+K]^+^, with heterolytic cleavage fragments forming product ions at *m/z* 267 and 269 and a homolytic cleavage fragment at *m/z* 268 (Figure 3H). We also detected and localized *in situ* salicin (*m/z* 325.072 [M+K]^+^) and salicortin (*m/z* 463.102 [M+K]^+^ (Figure S3A–C), with the same spatial distributions as for tremuloidin (Figure S4). Additionally, both phenolic glycosides are highly abundant in leaf of the hybrid poplar transformed with an empty vector (Figure S3C), while less abundant in the *RhPAAS/PAR1* and *PhPAAS/PAR1* transgenic poplar leaves.

## Discussion

This study focused on the transformation of hybrid poplar with the two enzymes involved in the PEA biosynthetic pathway as a potential means of systematically optimizing its production and ultimately further developing it in future as a platform for producing specialty/commodity chemicals. Interestingly, although functions of PAAS and PAR enzymes have been individually reported [8], [9], to our knowledge there were no reports of the effect of their combined expression when their encoding genes were engineered in tandem into transgenic plants. Thus, the potential use of hybrid poplar as a bioreactor for production of PEA and its derivatives (specialty chemicals of high value) was of considerable interest. Initially, the lines we generated had accumulation levels reaching 0.05% in fresh weight for PEA in leaf tissue (Figure 2A) at 7 weeks growth/development *versus* levels reaching 0.3% and 3–4% dry weight in stems and leaves of four-month-old plants for PEA-Glc, respectively (Figure 2C). It will be of considerable interest to establish what changes in their levels occur at different stages in the growing season, and as secondary stem thickening progresses from 2–5 years growth/development.

Comparison between plants transformed with empty vector and those expressing *RhPAAS* and *PAR1*, using untargeted metabolic profiling, also demonstrated a significantly increased accumulation of several putative PEA derivatives (Table S2). These metabolites, in addition to the most abundant PEA-Glc, are more hydrophilic, stable and non-volatile, when compared to PEA; indeed, all of these can perhaps increasingly act as a sink for PEA production. That is, the relatively toxic PEA produced by transgenes under control of the CaMV 35S promoter is apparently largely sequestered via glycosylation, or by other covalent linkages. In this way, this perhaps can lead to significantly higher carbon flux through the pathway with no observable detrimental effects to physiology and morphology. Glycosylation is a known storage mechanism and can be utilized for detoxification of various secondary metabolites [24]. We had thus correctly hypothesized that it might also be utilized for overproduction of “foreign” chemicals *in planta*. Indeed, sequestration by glycosylation (or other forms of derivatization) may be of crucial importance for the use of poplar (and probably other tree species) as bioreactors for production of specialty chemicals, i.e., given the ability of glycosylation in detoxifying such products and in reducing their volatility, thereby decreasing potential detrimental effects while leading to higher carbon flux levels. Indeed, the lack of any noticeable phenotypic alterations in trees up to several months old, despite considerable PEA-Glc content, tends to support this notion.

A second important effect noted was the decrease in accumulation of other well-known poplar phenylpropanoids (e.g., flavonoids etc.) that were readily identified based on their mass spectrometry data and literature values [20], [25]–[27] (Table S2). The reduction in the levels of these metabolites possibly result from carbon flux diversion and point to phenylalanine pool size as a limiting factor that could be addressed in future efforts to increase PEA production levels.

**Figure 4:**
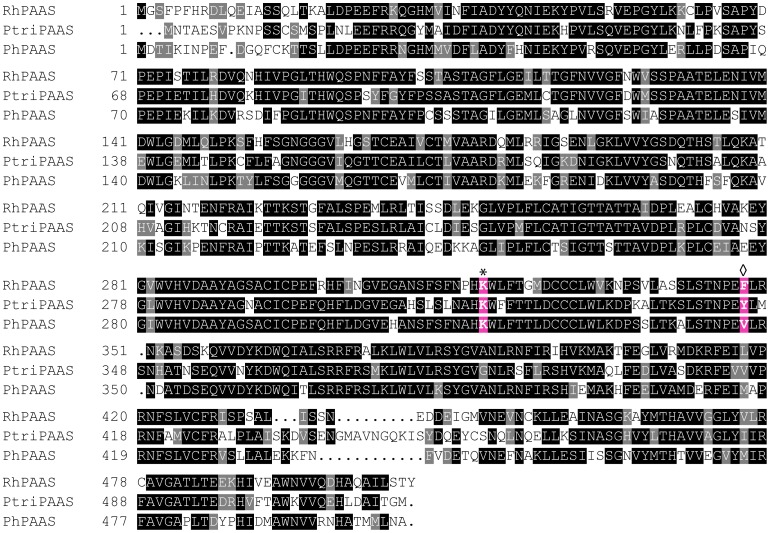
Amino acid sequence alignment for plant phenylacetaldehyde synthases. RhPAAS, *Rosa hybrida* cv. Fragrant Cloud (GenBank accession number DQ192639); PtriPAAS, *Populus trichocarpa* (EEE86177.1); PhPAAS, *Petunia hybrida* cv. Mitchell (DQ243784). This alignment was produced using the MultAlin software program [28]. Dots indicate gaps introduced into sequences to maximize alignments. The alignment was shaded using BoxShade Version 3.21 software program (Human Genome Sequencing Center, Houston,TX). Residues shaded in black indicate amino acids identical in at least two of the three sequences compared; gray shaded residues indicate matches that have similar properties. * conserved (pyridoxal 5′-phosphate)-binding residue at position 319 [9], [29]. ◊ Site of Tyr^332^ in pig kidney 3,4-dihydroxyphenylalanine decarboxylase that, when mutated to a Phe, caused the enzyme to show oxidative decarboxylation activity [30]. This same residue in RhPAAS is Phe, whereas in PhPAAS it is a Val.

Interestingly, there were large differences noted for production of PEA and PEA-Glc (Figure 2) in hybrid poplar expressing *RhPAAS* relative to *PhPAAS*. In this respect, relative expression levels of PAAS for both constructs were evaluated (Figure S2) and it was determined that the petunia derived gene had (apparent) lower transcript levels and this could perhaps contribute for the lower PEA and PEA-Glc levels relative to the *RhPAAS*. In addition though, a comparison of the amino acid sequences was conducted to examine if any other underlying factors possibly contributed to differences in the levels noted. Alignment of the PAAS homologues (using the MultAlin [28] software program) from various species, including rose, petunia, and poplar thus established that, while all contain the necessary pyridoxal 5′-phosphate-binding lysine residue [9], [29] (Figure 4, RhPAAS residue 319 indicated by asterisk), only the rose PAAS amino acid sequence contains the phenylalanine residue (Phe^348^) known to be associated with oxidative decarboxylation activity [9]. The role of this critical residue was previously discovered by Bertoldi and co-workers [30] who demonstrated that mutation of a single tyrosine to a phenylalanine residue in pig kidney 3,4-dihydroxyphenylalanine decarboxylase resulted in PAAS-like oxidative decarboxylation activity. Our results therefore indicate that, among the *PAAS* genes studied thus far, the phenylalanine residue at that position may also be needed for the increased PAAS activity noted in the rose PAAS homolog. Furthermore, hybrid poplar contains a PAAS homolog (PtriPAAS), sharing ∼66% amino acid identity to RhPAAS. It also has a tyrosine residue at the position equivalent to Tyr^332^ in the pig kidney 3,4-dihydroxyphenylalanine decarboxylase studied [30], instead of phenylalanine (Figure 4). Together, the distinct expression levels and the amino acid difference may be responsible for the differences observed in PEA/PEA-Glc production in both the *RhPAAS/PAR1* and *PhPAAS/PAR1* lines (Figure 2).

It was therefore next instructive to investigate whether transforming hybrid poplar with the tomato *PAR* gene might by itself also increase PEA/PEA-Glc levels. In this regard, an NCBI GenBank database BLAST search indicated presence of two *P. trichocarpa* cinnamoyl CoA reductase-like proteins having ∼76% amino acid identity (accession numbers XP_002314079.1 and XP_002298395.1, respectively) to LePAR1, and ∼84% identity to a rose PAR [31], perhaps suggesting that the entire pathway to PEA was in place in hybrid poplar. We thus transformed the hybrid poplar with *PAR* alone, but this gave no significant increase in PEA/PEA-Glc levels (data not shown). Accordingly, the lack of additional PEA production in poplar transformed with the *PAR* cDNA alone suggests that the native poplar PAAS homolog did not possess any significant level of PAAS activity. However, in this case, the poplar homolog was driven by its native promoter and not over-expressed by the CaMV 35S promoter as for *RhPAAS* or *PhPAAS*.

MALDI-TOF IMS successfully mapped the spatial distribution and localization of PEA-Glc in the transgenic hybrid poplar leaves. While it did not indicate PEA-Glc was readily detectable in the midrib, evaluation and analyses of solubles of isolated midribs established its presence there as well. Apparently, the laser ablation technologies at present are not very effective with this cell (wall) type. In addition, the high potassium adduct ion intensity observed in these tissues may provisionally be explained by the high concentration of potassium salts in the analyzed tissues, with PEA-Glc localized in the leaf midrib and lateral veins of *RhPAAS/PAR1* transformants. PEA-Glc was also localized in the leaf of transgenic poplar expressing *PhPAAS/PAR1*, but at a lower abundance (*circa* 9 fold decrease in signal intensity counts (Figure S3) compared to the transgenic poplar leaf harboring *RhPAAS/PAR1*. It was not detected though in hybrid poplar transformed with an empty vector.

*Populus tremula* leaves are rich in phenolic glycosides, including tremuloidin, salicin and salicortin [21]–[23]. We thus also used MALDI-TOF IMS to define their spatial distribution and localization *in situ*. The potassium adduct ions of tremuloidin (*m/z* 429.096, [M+K]^+^), salicin (*m/z* 325.072, [M+K]^+^), and salicortin (*m/z* 463.102, [M+K]^+^) were readily detected, and they were distributed in the leaf lamina and mainly localized in the vasculature. To the best of our knowledge, these data provide the first evidence of localization of these glycosides *in planta*. Aside from mapping their spatial distribution and localization, our work also provided information on their relative abundance in leaves. They were highly abundant in the leaf of hybrid poplar transformed with an empty vector, but apparently less abundant in leaves harboring *RhPAAS/PhPAAS* and *PAR1* genes, i.e., opposite to what was found for PEA-Glc (Figure 3, Figure S3 and S4). This result suggests that carbon metabolic flux (predominantly leaf tissue) is re-directed towards PEA/PEA-Glc biosynthesis and away from the phenolic glycosides. This is an important finding, as it suggests we can begin to tailor carbon flux to specific specialty/commodity chemicals of interest, such as PEA, from competing biochemical pathways, including that of lignification in the stems.

## Conclusions

Woody plants can produce up to 60% of phenylpropanoid derived substances in their heartwood tissues [13], suggesting that various trees (such as with hybrid poplar) on biotechnological manipulation may serve in future as a platform for commodity/specialty chemical production. In this study, we thus demonstrated that even at an early growth/developmental stage of transgenic poplar, levels of PEA-Glc can already amount to ∼4% in leaf tissue without perceptible detriment to growth and development. Future field trials currently underway will next establish the yields of PEA-Glc in both stem and leaf tissue as the trees mature over a 5-year time period, as well as effects on other parameters, such as lignin content and overall performance of transgenic plants. Furthermore, with proof-of-concept in place, additional future work will now be extended towards other biotechnological strategies aimed at attaining much higher levels of PEA (Glc) in fast growing poplar woody stem tissue with a goal of up to 40–50% by weight. Engineering hybrid poplar to produce compounds such as PEA thus now offers the opportunity to economically produce needed amounts of industrial chemicals using rapidly growing woody biomass as a sustainable platform. This in turn could contribute to sustainable solutions to competitively generate domestic specialty/commodity chemicals as petroleum replacements or substitutes.

## Supporting Information

Figure S1 **Time-course of 2-phenylethanol and phenylacetaldehyde accumulation in cultures of** ***E. coli*** **transformed with** ***PhPAAS/PAR1*** **and** ***RhPAAS/PAR1*** **.** Error bars represent standard deviation, with n = 3.
(TIF)

Figure S2 **Relative expression levels of** ***PAAS*** **(A) and PAR (B) in** ***PhPAAS/PAR1*** **,** ***RhPAAS/PAR1*** **and empty vector transformed hybrid poplar.** The lines analyzed have distinct but somewhat opposite transcript levels of both *PAAS* and *PAR1*.
(TIF)

Figure S3 **MALDI-TOF IMS generated spectra showing relative abundances of the metabolites at** ***m/z*** **range 100 to 700 Da.** (A) *RhPAAS/PAR1*, (B) *PhPAAS/PAR1* and (C) hybrid poplar empty vector (control) leaves using 2, 5-dihydroxybenzoic acid (DHB) as matrix. The data were acquired in positive mode using a resolution mode in the range *m/z* 100 to 700 Da with spatial resolution of 50 µm (leaves) at laser energy of 250 and firing rate of 1000 Hz. Metabolites identified were color coded: red – PEA-Glc; orange – putative salicin; green – tremuloidin; and pink – putative salicortin.
(TIF)

Figure S4 **Spatial distribution of salicortin and salicin in leaf tissues of transgenic poplar as analyzed by MALDI imaging mass spectrometry.** MALDI image and ion intensity map of salicortin (*m/z* 463.10 [M+K]^+^) and salicin (*m/z* 325.07 [M+K]^+^) in hybrid white poplar transformed with: *RhPAAS*/*PAR1* (A and D), *PhPAAS*/*PAR1* (B and E), and an empty vector (C and F). Data was obtained with spatial resolution of 50 µm and using 2,5-dihydroxybenzoic acid as matrix. Note: The midrib was resistant to laser ablation and thus detection of metabolites was apparently not achieved fully in this region.
(TIF)

Table S1 Primers used for PCR amplifications.
(PDF)

Table S2 PEA-Glc, other putative PEA derivatives and flavonoids detected in leaf tissue from PEA-producing hybrid poplars.
(PDF)
