# Supplementary figures and images for: Transgenic Hybrid Poplar for Sustainable and Scalable Production of the Commodity/Specialty Chemical, 2-Phenylethanol
Source: PLoS One. 2013 Dec 26;8(12):e83169. doi: 10.1371/journal.pone.0083169 (PMC3873308; doi:10.1371/journal.pone.0083169)

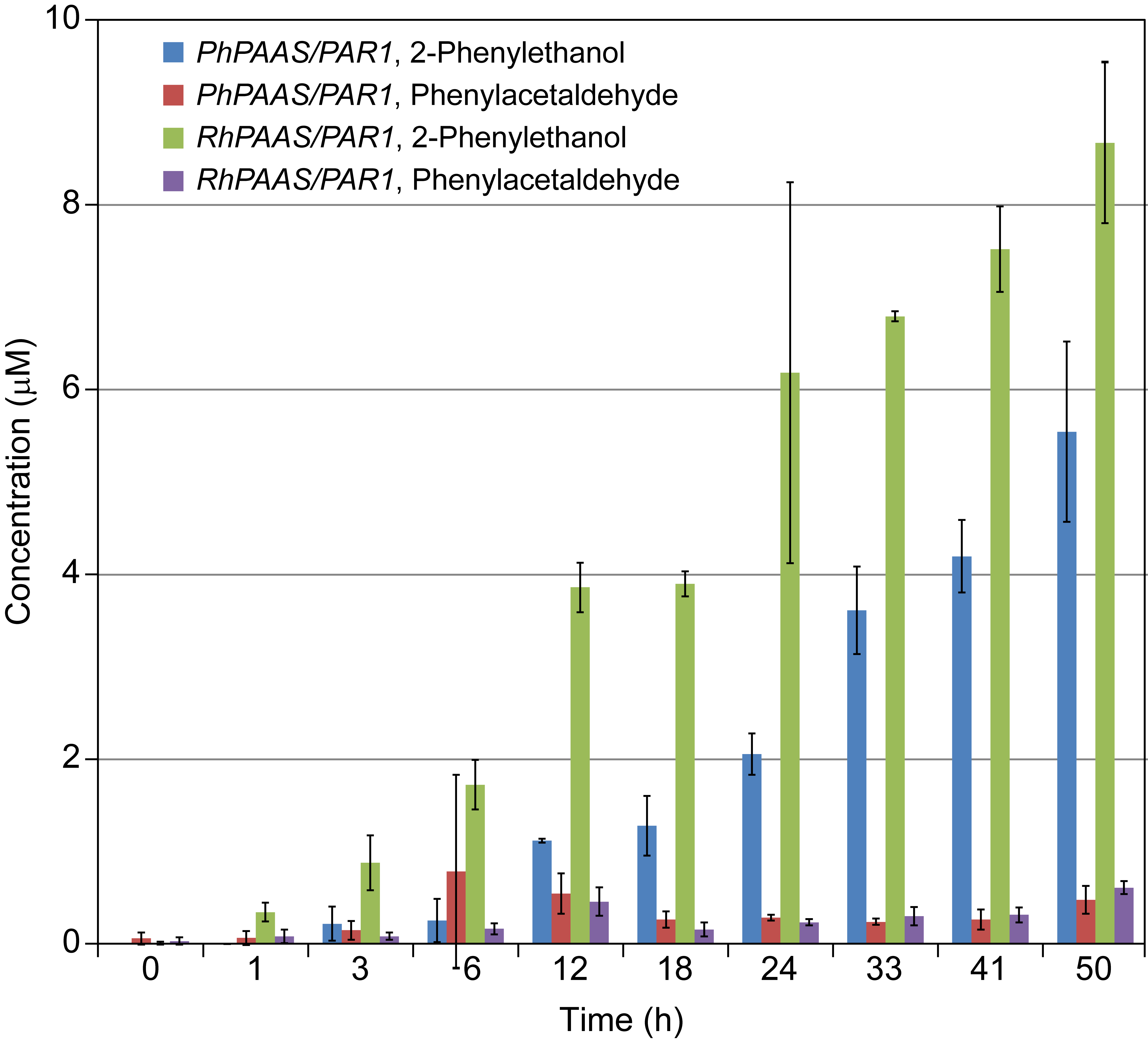

Supplement: Figure S1 — Time-course of 2-phenylethanol and phenylacetaldehyde accumulation in cultures of E. coli transformed with PhPAAS/PAR1 and RhPAAS/PAR1 . Error bars represent standard deviation, with n = 3. (TIF) [file pone.0083169.s001.tif]

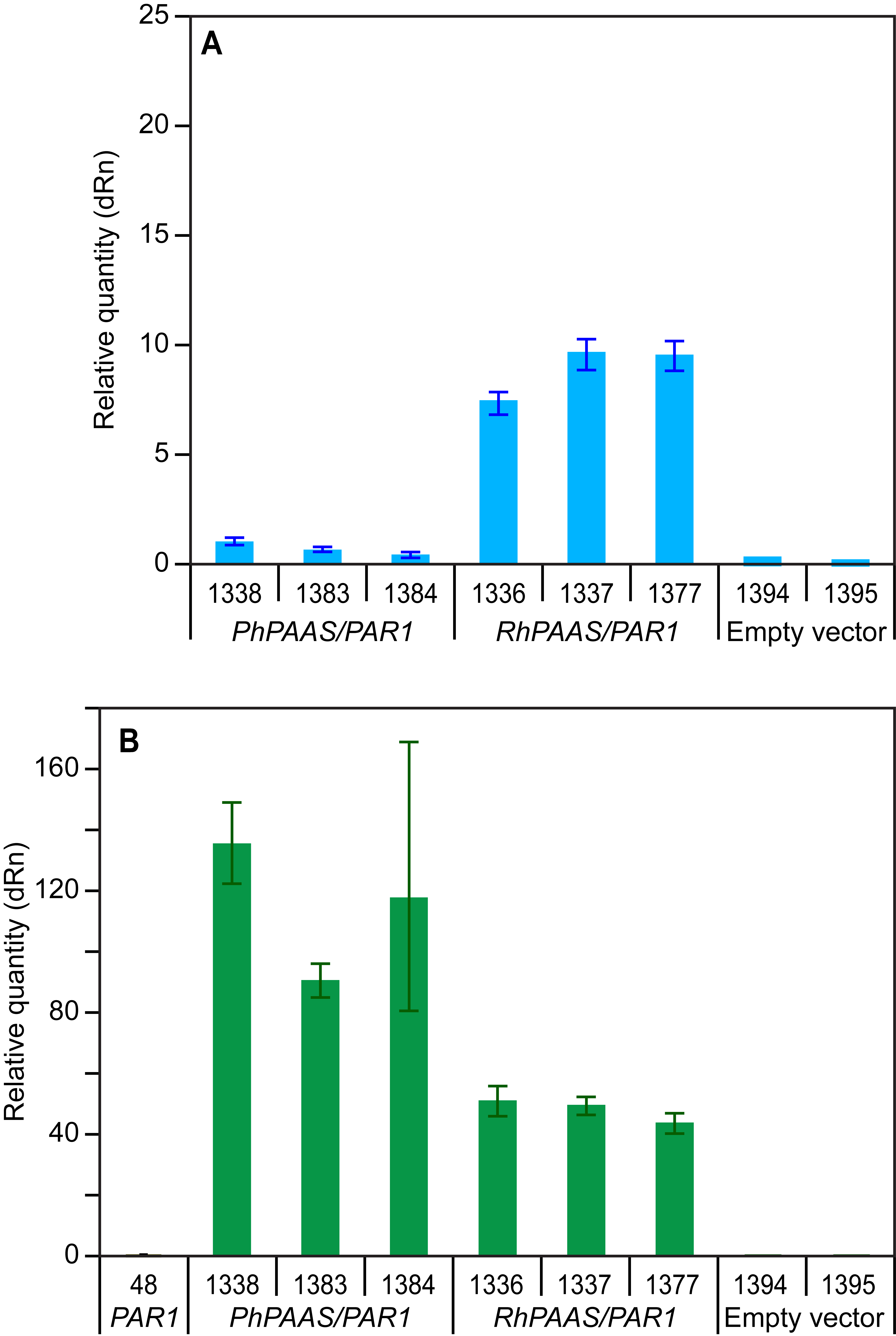

Supplement: Figure S2 — Relative expression levels of PAAS (A) and PAR (B) in PhPAAS/PAR1 , RhPAAS/PAR1 and empty vector transformed hybrid poplar. The lines analyzed have distinct but somewhat opposite transcript levels of both PAAS and PAR1. (TIF) [file pone.0083169.s002.tif]

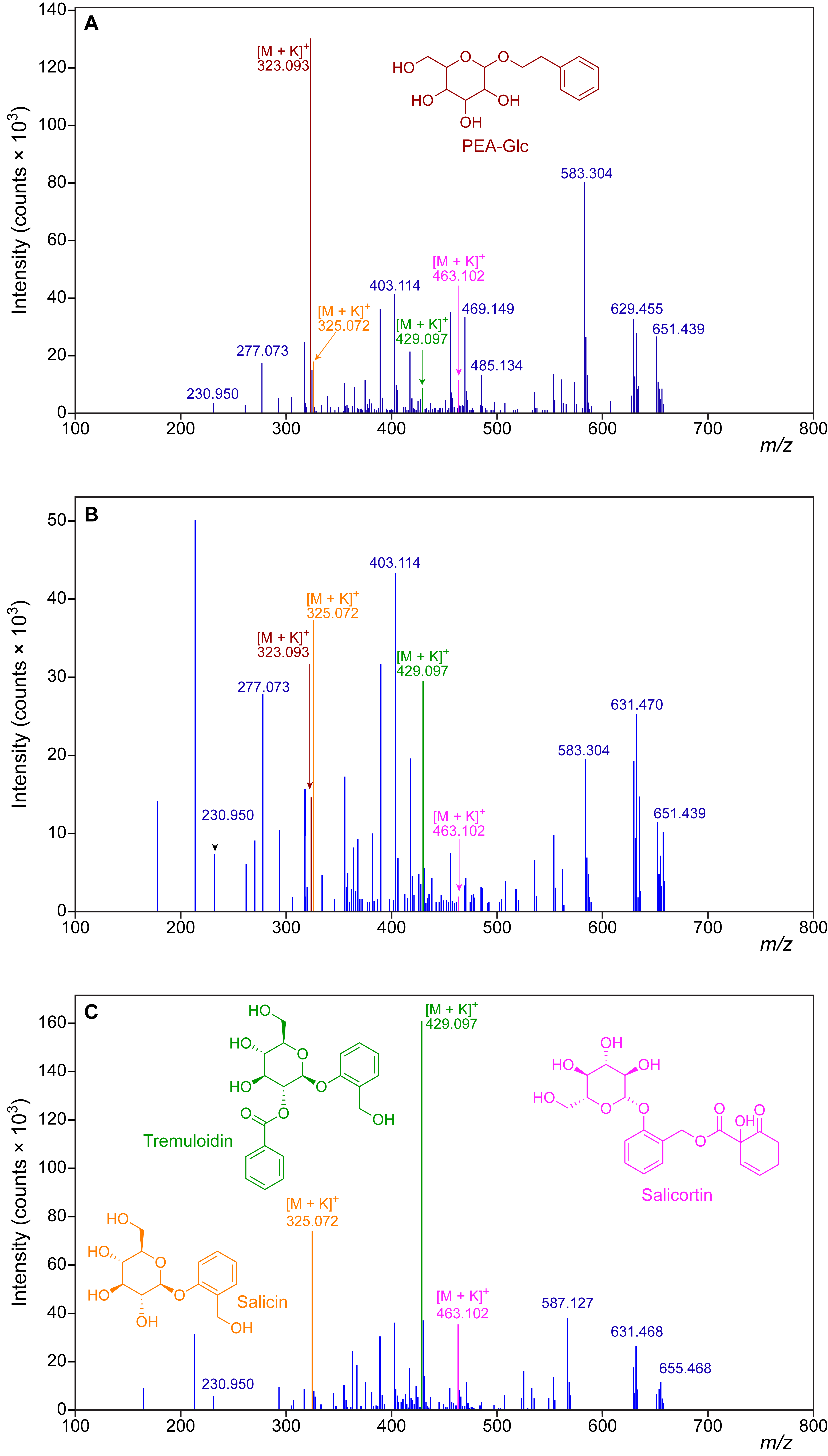

Supplement: Figure S3 — MALDI-TOF IMS generated spectra showing relative abundances of the metabolites at m/z range 100 to 700 Da. (A) RhPAAS/PAR1, (B) PhPAAS/PAR1 and (C) hybrid poplar empty vector (control) leaves using 2, 5-dihydroxybenzoic acid (DHB) as matrix. The data were acquired in positive mode using a resolution mode in the range m/z 100 to 700 Da with spatial resolution of 50 µm (leaves) at laser energy of 250 and firing rate of 1000 Hz. Metabolites identified were color coded: red – PEA-Glc; orange – putative salicin; green – tremuloidin; and pink – putative salicortin. (TIF) [file pone.0083169.s003.tif]

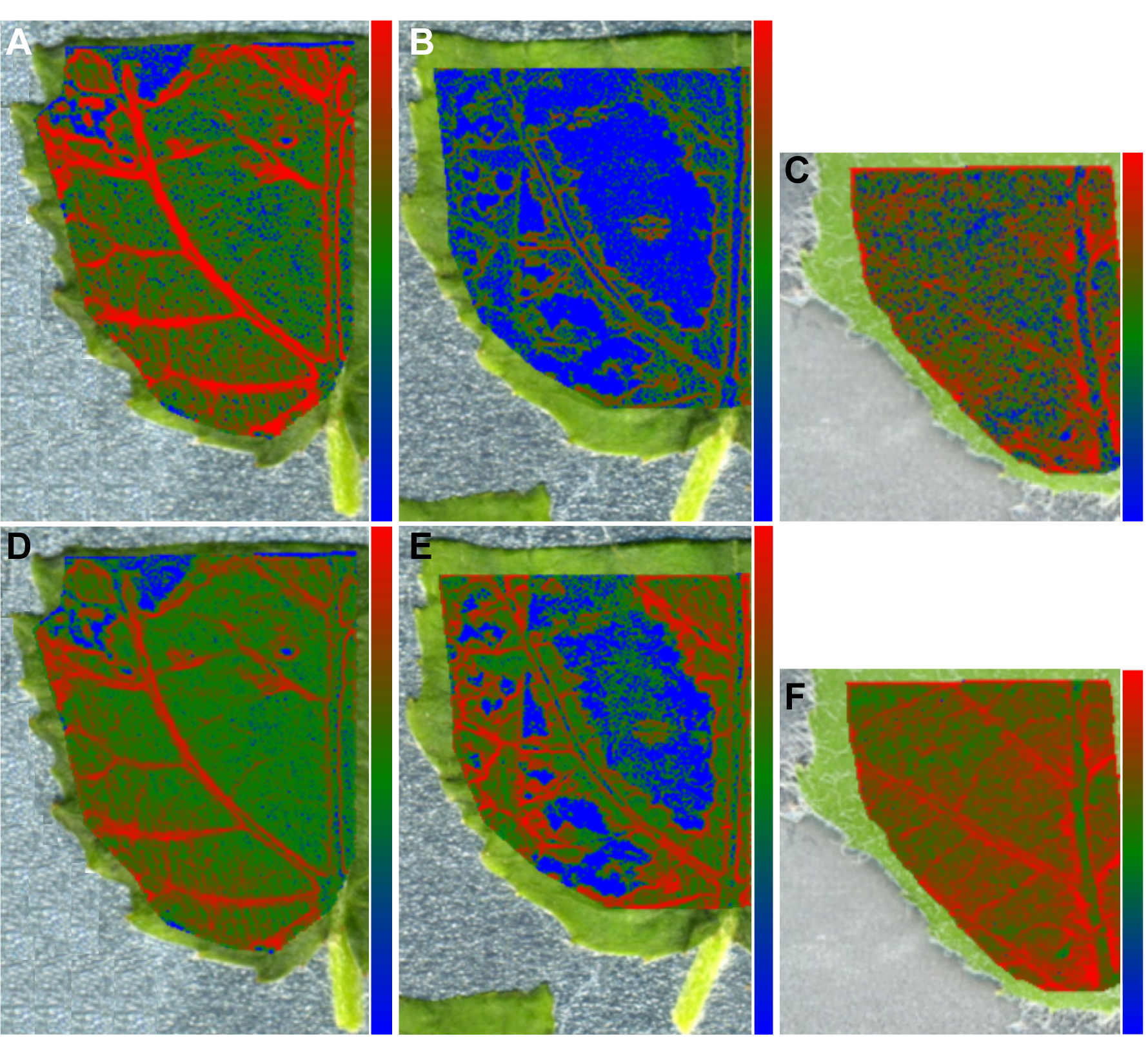

Supplement: Figure S4 — Spatial distribution of salicortin and salicin in leaf tissues of transgenic poplar as analyzed by MALDI imaging mass spectrometry. MALDI image and ion intensity map of salicortin (m/z 463.10 [M+K]+) and salicin (m/z 325.07 [M+K]+) in hybrid white poplar transformed with: RhPAAS/PAR1 (A and D), PhPAAS/PAR1 (B and E), and an empty vector (C and F). Data was obtained with spatial resolution of 50 µm and using 2,5-dihydroxybenzoic acid as matrix. Note: The midrib was resistant to laser ablation and thus detection of metabolites was apparently not achieved fully in this region. (TIF) [file pone.0083169.s004.tif]
