# Supplementary material for: Transgenic Hybrid Poplar for Sustainable and Scalable Production of the Commodity/Specialty Chemical, 2-Phenylethanol
Source: PLoS One. 2013 Dec 26;8(12):e83169. doi: 10.1371/journal.pone.0083169 (PMC3873308; doi:10.1371/journal.pone.0083169)
Supplement: Table S1 — Primers used for PCR amplifications. (PDF) [file pone.0083169.s005.pdf]

Table S1. Primers used for PCR amplifications

Primer name	Sequence
RhFCPAAS-F	5′-CACCATGGGTAGCTTCCCATTCCAC-3′
RhFCPAAS-R	5′-TCAATACGTGCTGAGGATTGC-3′
RhFCPAAS-BspH I-DUET-F	5′- (CACC)TCATGATGGGTAGCTTCCCATTCCACAGG-3′
RhFCPAAS-Not I-DUET-R	5′-GCGGCCGCTCAATACGTGCTGAGGATTG-3′
RhFCPAAS-Xho I-F	5′-CACCCTCGAGATGGGTAGCTTCCCATTCC-3′
RhFCPAAS-Bgl II-R	5′-AGATCTTCAATACGTGCTGAGGATTGCTTG-3′
(PAAS)-attB5-p35S-F	5′-GGGGACAACTTTGTATACAAAAGTTGTCGACGAATTAATT CCAATCCCACA-3′
(PAAS)-attB2-tOCS-R	5′-GGGGACCACTTTGTACAAGAAAGCTGGGTAAGATTTAGG TGACACTATAGAATATGCATCACTAGTAAGCTAGC-3′
RhFCPAAS-QPCR-F	5′-AAGAATACGGCGTGTGGGTTCA-3′
RhFCPAAS-QPCR-R	5′-GGCAACAGCAATCCATTCCAGT-3′
PhMPAAS-F	5′-ATGGATACTATCAAAATCAACCCAGAATTT-3′
PhMPAAS-R	5′-CTACGCATTCAGCATCATAGTTGCATGGTT-3′
PhMPAAS-BspH I-DUET-F	5′- (CACC)TCATGATGGATACTATCAAAATCAACCCAG-3′
PhMPAAS-Sac I-DUET-R	5′-GAGCTCCTACGCATTCAGCATCATAG-3′
PhMPAAS-Xho I-F	5′-CACCCTCGAGATGGATACTATCAAAATCAACCCAG-3′
PhMPAAS-Bgl II-R	5′-AGATCTCTACGCATTCAGCATCATAGTTGC-3′
PhMPAAS-QPCR-F	5′-GTGCTGGTCTTAACGTGGTTGG-3′
PhMPAAS-QPCR-R	5′-ACACCACCACCACCACCAGAA-3′
LePAR1-F	5′-CACCATGAGTGTGACAGCGAAAACA-3′
LePAR1-R	5′-TTACATAGAAGATGAACCTCC-3′
LePAR1-Nde I-DUET-F	5′-CATATGAGTGTGACAGCGAAAACAGTGTGTG-3′
LePAR1-Xho I-DUET-R	5′-CTCGAGTTACATAGAAGATGAACCTCCAAA-3′
(LePAR1)-attB1-p35S-F	5′-GGGGACAAGTTTGTACAAAAAAGCAGGCTCTAGAGCCAA GCTGATCTCCT-3′
(LePAR1)-attB5r-t35S-R	5′-GGGGACAACTTTTGTATACAAAGTTGTCTAGAGGGCCCGA CGTCGCAT-3′
LePAR1-QPCR-F	5′-TGTGAAGGCTTCTGTTCGTG-3′
LePAR1-QPCR-R	5′-GCACATGACCCGAGAAGATT-3′
PtaACTIN-QPCR-F	5′-GGTCCTCTTCCAACCTTCAA
PtaACTIN-QPCR-R	5′-TCCTGGGAACATAGTTGAACC-3′
PAAS-RhFC/PhM-QPCR-FOR	5′-CATTGAAGCTGTGGCTTGTG-3′
PAAS-RhFC/PhM-QPCR-REV	5′-CAACCACAGCATGAGTCATGTA-3′
