# Supplementary material for: Transgenic Hybrid Poplar for Sustainable and Scalable Production of the Commodity/Specialty Chemical, 2-Phenylethanol
Source: PLoS One. 2013 Dec 26;8(12):e83169. doi: 10.1371/journal.pone.0083169 (PMC3873308; doi:10.1371/journal.pone.0083169)
Supplement: Table S2 — PEA-Glc, other putative PEA derivatives and flavonoids detected in leaf tissue from PEA-producing hybrid poplars. (PDF) [file pone.0083169.s006.pdf]

Table S2. PEA-Glc, other putative PEA derivatives and flavonoids detected in leaf tissue from PEA-producing hybrid poplars. All compounds except for PEA-Glc were tentatively identified based on MS data. Glc = glucose; Rha = rhamnose, Xyl = xylose and Shik = shikimate. Fold increase/decrease in *RhPAAS/PAR1* in comparison with control (tree transformed with empty vector). P values derived from Welch's two-sample t-test are shown in brackets.

**Authentic** and/or putative metabolites	Formula	Measured mass	Calculated mass	Error (ppm)	Fold increase (P) in leaf	Fold increase (P) in stem	Other ions observed (ion, error)
**PEA-Glc**	$C_{14}H_{21}O_6$	285.1340	285.1338	0.7	722 (0.01)	77 (0.03)	302.1604 ($[M+NH_4]^+$, 0.1 ppm) ; 569.2605 ($[2M+H]^+$, 1.2 ppm)
PEA-Glc-Glc	$C_{20}H_{31}O_{11}$	447.1862	447.1866	0.8	932 (0.06)	167 (0.04)	n.o.[a]
PEA-Glc-Rha	$C_{20}H_{31}O_{10}$	431.1917	431.1917	0	25 (0.001)	30 (0.01)	861.3739 ($[2M+H]^+$, 2.0 ppm); 453.1732 ($[M+Na]^+$, 1 ppm); 309.1188 ($[Glc\text{-}Rha\text{-}H_2O+H]^+$, 0.8 ppm).
PEA-Shik-Rha	$C_{21}H_{28}O_9Na$	447.1626	447.1631	1.1	216 (0.007)	36 (0.005)	447.1626 ($[M+Na]^+$, 1.2 ppm); 442.2027 ($[M+NH_4]^+$, 0.7 ppm); 105.0702 ($[M+H\text{-}shik\text{-}rha]^+$, 2.0 ppm)
PEA-Glc-Xyl	$C_{19}H_{29}O_{10}$	417.1763	417.1761	0.5	24 (0.009)	260 (0.009)	439.1573 ($[M+Na]^+$, 1.6 ppm); 434.2015 ($[M+NH_4]^+$, 2.6 ppm) 295.1026 ($[M+H\text{-}phenylethanol]^+$, 1 ppm)

**Known[1-4]** and/or putative metabolites	Formula	Measured Mass	Calculated Mass	Error (ppm)	Fold decrease (P) in leaf	Fold decrease (P) in stem	Other ions observed (ion, error)
Caffeic acid-Glc	$C_{15}H_{19}O_9$	343.1031	343.1029	0.6	33 (0.001)	4 (0.02)	n.o.[a]
**Benzoic acid**	$C_7H_7O_2$	123.0447	123.0446	0.8	45 (0.0002)	8 (0.002)	n.o.
Flavan-3-ol-Glc	$C_{22}H_{26}O_{11}Na$	489.1373	489.1373	0.0	24 (0.016)	2 (0.11)	n.o.
**Rhamnetin-Glc**	$C_{22}H_{23}O_{12}$	479.1179	479.1189	2.1	17 (0.0005)	n.o.	317.0656 ($[M\text{-}Glc+H]^+$, 1.6 ppm)
**Kaempferol**	$C_{15}H_{11}O_6$	287.0555	287.0556	0.3	3 (0.009)	n.o.	n.o.
Kaempferol-rutinoside	$C_{27}H_{31}O_{15}$	595.1664	595.1663	0.2	9 (0.004)	n.o.	449.1078 ($[M\text{-}Rha+H]^+$, 1.3 ppm)
**Quercetin**	$C_{25}H_{11}O_7$	303.0504	303.0505	0.3	9 (0.01)	n.o.	163.0388 ($C_9H_7O_3$, 4 ppm)
**Rutin**	$C_{27}H_{31}O_{16}$	611.1613	611.1612	0.1	6 (0.01)	n.o.	465.1034 ($[M\text{-}Rha+H]^+$, 0.2 ppm)
Quercetin-Glc-Xyl	$C_{26}H_{29}O_{16}$	597.1462	597.1455	1	9 (0.0006)	n.o.	465.1042 ($[M\text{-}Xyl+H]^+$, 1.9 ppm); 303.0504 ($[M\text{-}Xyl\text{-}Glc+H]^+$, 0.2 ppm)
Penta-methylated myricetin	$C_{20}H_{21}O_8$	389.1233	389.1236	0.9	4 (0.05)	3 (0.04)	371.1127 ($[M\text{-}Glc+H]^+$, 1.0 ppm)
Umbelliferone-Gal	$C_{15}H_{17}O_7$	309.0973	309.0974	0.4	3 (0.0003)	n.o	147.0446 ($[M\text{-}Gal+H]^+$, 0 ppm)
**Tremuloidin**	$C_{20}H_{23}O_8Na$	413.1211	413.1212	0.4	1.3 (0.2)	n.o.	Tremuloidin

[a]n.o.: not observed

1. Tsai C-J, Harding SA, Tschaplinski TJ, Lindroth RL, Yuan Y (2006) Genome-wide analysis of the structural genes regulating defense phenylpropanoid metabolism in *Populus*. New Phytologist 172: 47-62.
2. Crawford DJ (1974) A morphological and chemical study of *Populus acuminata* Rydberg. Brittonia 26: 74-89.
3. Morreel K, Goeminne G, Storme V, Sterck L, Ralph J, et al. (2006) Genetical metabolomics of flavonoid biosynthesis in *Populus*: A case study. The Plant Journal 47: 224-237.
4. Warren JM, Bassman JH, Fellman JK, Mattinson DS, Eigenbrode S (2003) Ultraviolet-B radiation alters phenolic salicylate and flavonoid composition of *Populus trichocarpa* leaves. Tree Physiology 23: 527-535.
